# Online SFRA for Reliability of Power Systems: Characterization of a Batch of Healthy and Damaged Induction Motors for Predictive Maintenance

**DOI:** 10.3390/s23052583

**Published:** 2023-02-26

**Authors:** Giovanni Bucci, Fabrizio Ciancetta, Andrea Fioravanti, Edoardo Fiorucci, Simone Mari, Andrea Silvestri

**Affiliations:** Dipartimento di Ingegneria Industriale e dell’Informazione e di Economia, Università dell’Aquila, 67100 L’Aquila, Italy

**Keywords:** predictive maintenance, induction motors, industrial measurements, measurement system, frequency response, custom instrumentation

## Abstract

Asynchronous motors represent a large percentage of motors used in the electrical industry. Suitable predictive maintenance techniques are strongly required when these motors are critical in their operations. Continuous non-invasive monitoring techniques can be investigated to avoid the disconnection of the motors under test and service interruption. This paper proposes an innovative predictive monitoring system based on the online sweep frequency response analysis (SFRA) technique. The testing system applies variable frequency sinusoidal signals to the motors and then acquires and processes the applied and response signals in the frequency domain. In the literature, SFRA has been applied to power transformers and electric motors switched off and disconnected from the main grid. The approach described in this work is innovative. Coupling circuits allow for the injection and acquisition of the signals, while grids feed the motors. A comparison between the transfer functions (TFs) of healthy motors and those with slight damage was performed with a batch of 1.5 kW, four-pole induction motors to investigate the technique’s performance. The results show that the online SFRA could be of interest for monitoring induction motors’ health conditions, especially for mission-critical and safety-critical applications. The overall cost of the whole testing system, including the coupling filters and cables, is less than EUR 400.

## 1. Introduction

Progress in the use of electrical systems for efficiency and the development of intelligent systems in the industrial sector entails an increasing role of electric motors. Although the purchase cost of grid-powered asynchronous motors is extremely low, it is nevertheless interesting to investigate their state of health during normal operating conditions [1,2,3,4,5,6,7,8,9,10,11,12]. This is because the anomalies reported by electric motors during their normal operation can evolve into serious failures that cause process disservices and numerous safety problems.

For example, they are adopted in power systems for cooling high-power machines, such as high-voltage power transformers of HVDC, static synchronous compensation, and static reactive power compensation applications [13]. Moreover, their faults can also involve power quality phenomena, disturbing neighboring devices [14,15,16].

The anomalies affecting an induction motor are different and often not identifiable by traditional condition monitoring systems. Most faults are located in the stator windings, initially causing effects on the rotation and then evolving into worse effects.

The importance of fault diagnosis and the specific problem of identifying and monitoring faults in induction motors have been extensively discussed in the literature [1,5]. To date, grid anomaly detection systems have been based either on the measurement of mechanical vibrations and acoustic emissions or the processing of thermographic measurements through image processing software. In addition, the measurement of electrical parameters allows for the realization of anomaly detection and fault diagnosis systems; for example, the evaluation of the negative sequence impedance and the detection of voltage misalignments [12] were widely proposed techniques in the past. The cited techniques allow for the realization of systems of reduced complexity since the computational resources are modest. On the other hand, these systems do not allow for the detection of anomalies in a short time [17].

While the choice of mechanical, thermal, or electrical parameters to be monitored is fundamental, the design of an anomaly detection system cannot be separated from an efficient and effective processing section. To this end, approaches based on the analysis of the waveforms of the stator winding current have been proposed over the years, carried out by adopting different methods: spectral or wavelet analysis, fuzzy logic, and neural networks [9,11,18,19,20,21,22,23].

In addition to the previously mentioned non-invasive techniques, several authors have focused on designing diagnostic systems based on ad hoc stator tooth coils [24] or Hall-effect transducers [25] to overcome some drawbacks of traditional diagnostic techniques.

It is also possible to use online techniques based on the injection of test signals on the stator windings and the processing of the frequency response with different methodologies, such as, for example, the online sweep frequency response analysis (SFRA). The main advantage of online techniques is avoiding the motor’s deactivation due to the costs and actions required to stop complex and critical processes [26,27,28,29,30]. Another advantage of these techniques is the reduced cost of the measurement equipment.

However, these techniques are based on evaluating the variations in the characteristic curves of the motor. Changes that can occur over time due to normal aging or wear are considered initial symptoms of impending failures. Aging and wear cannot be determined a priori due to possible overloads, bearing damage, or even improper maintenance actions. Therefore, it is necessary to acquire information on a sufficient number of new motors under different operating conditions to experimentally verify these techniques’ performance. This allows for the definition of some reference curves to detect deviations and identify the possible causes of initial failure.

For this purpose, 10 new asynchronous motors from the same production batch were acquired and characterized with a dynamometer for nominal and variable load tests. The characteristics were achieved with a grid power supply, and without the interposition of transformers or other systems, to reproduce the normal operating conditions of grid-powered asynchronous motors. Furthermore, a passive dynamometer was used for these tests, without energy recovery, so as not to modify the electrical characteristics of the power supply network during the tests.

## 2. The Testing System

The SFRA measurement technique studied generally requires the injection of a sinusoidal signal between two terminals of an electrical winding. It is applied to both rotating electrical machines and transformers. The signal typically has an amplitude of a few voltages, with frequencies ranging from a few hertz to 1–2 MHz. The traditional SFRA technique is applied to non-powered devices, which are taken out of service during the test.

The proposed technique is instead performed online [30], performing measurements without interrupting the operation. The low-amplitude test signal is superimposed on the power signal and does not interfere with the normal operation of the equipment. In previous works [31,32,33], we have illustrated the results obtained by carrying out different campaigns of experimental measurements on various devices, using systems developed ad hoc to perform standard and online SFRA.

Grid-coupling filters were implemented to perform the SFRA online, suitably designed to operate as a bandpass from 2 kHz to 1.5 MHz. They were necessary to block the fundamental component at 50 Hz and the voltage harmonics up to 40° returning from the grid to the measurement system by allowing for the application and the acquisition of the signals in safe conditions. Both the SFRA signal generation and acquisition were performed with a Digilent Analog Discovery 2 NI Edition board with a BNC adapter [29,30,31,32] that was controlled by a PC with test software implemented in the NI LabVIEW Environment. This board operated as a two-channel oscilloscope with differential inputs, 14-bit resolution, a ±25 V input range, 30 MHz bandwidth, and a 100 MSample/s sampling frequency. In addition, the board embodies a two-channel arbitrary function generator with a ±5 V output range, 20 MHz bandwidth, and a 100 MSample/s sampling frequency. The total hardware cost of the entire test system, including the filters and cables, was less than EUR 400.

The proposed NI LabVIEW measurement software is an evolution of the version presented and described in [30]. The most critical goal when designing this proposed system was the reduction of the S/N due to the high electromagnetic noise generated by the motor under test, which can lead to several SFRA measurement problems.

**Figure 1:**
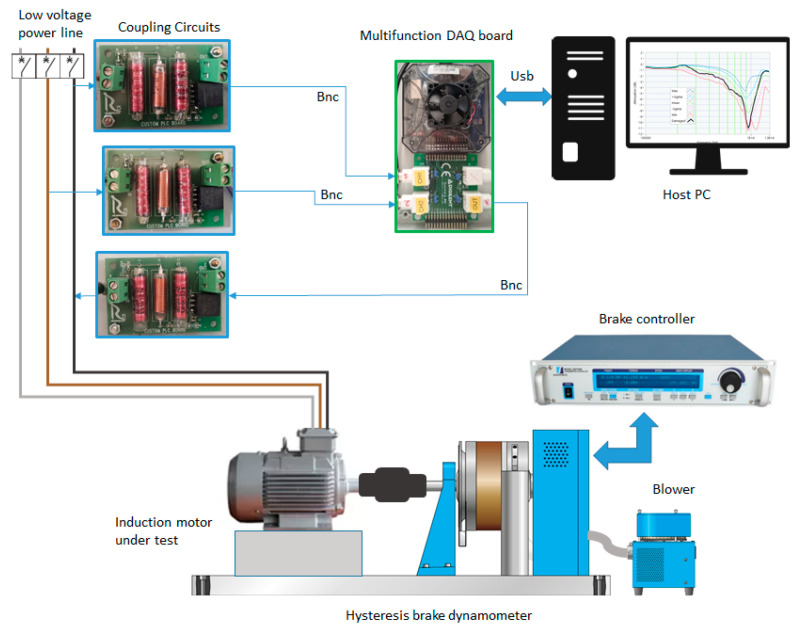
Block diagram of the experimental setup.

**Figure 2:**
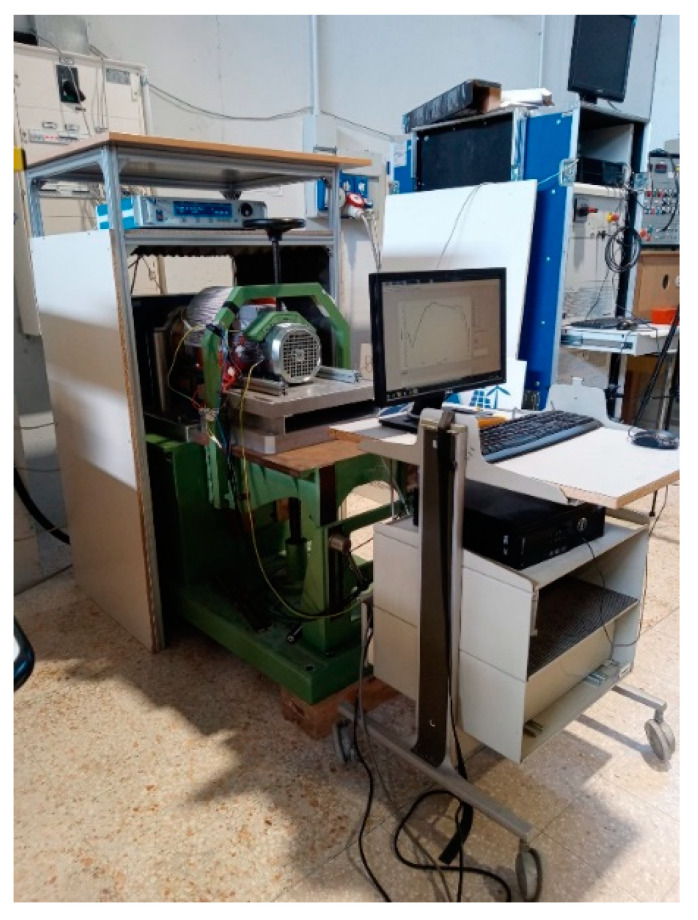
The testing bench.

The measurement procedure is based on the following steps: (1) simultaneous acquisition of the input Vin (with reference to the motor under test) and the output Vout signals; (2) application of the Hanning window to both signals; (3) processing of the fast Fourier transform (FFT) of the Vin and measurement of the amplitude and frequency (bin position) of the test signal; and (4) FFT processing of the Vout and identification of the component with the same bin position as the Vin. As previously mentioned, with the test bench, we reproduced the actual operating conditions of the asynchronous motor, feeding it from the grid without an inverter or energy recovery system, in order to not introduce disturbances to the power supply line, to avoid altering the results of the test. The load was carried out using a Magtrol HD 815 hysteresis dynamometer equipped with a Magtrol TM 108 torque and speed transducer. Furthermore, the brake was controlled with a Magtrol DSP 6001 unit, which also acquired torque and speed rotation (Figure 1 and Figure 2).

## 3. Characterization of a Set of Healthy Motors

The experimental tests were performed on a set of 10 new three-phase induction motors, model BE 90 LA4 produced by Bonfiglioli [33]. The rated features of the @50 Hz were: (1) voltage of 230/400 V Δ/Y, (2) current of 6.1/3.5 A Δ/Y; (3) power of 1.5 kW; (4) speed of 1430 rpm; (5) power factor of 0.74; (6) efficiency of 82.5% at 100% load, complying with IE2 according to IEC EN 60034; (7) insulation class F, IP 55. According to a more detailed analysis of the methods for execution of the tests, it is possible to note that the motor under test was connected in parallel to the power supply system, which consisted of cables, power transformers, and an upstream electrical network. The test signal was applied to both the motor under test and the supply system in the parallel connection. The diagnostic system therefore had to separate the effects of the motor from those relating to all other external devices. The SFRA of the grid node supplying the motor was the first be acquired.

**Figure 3:**
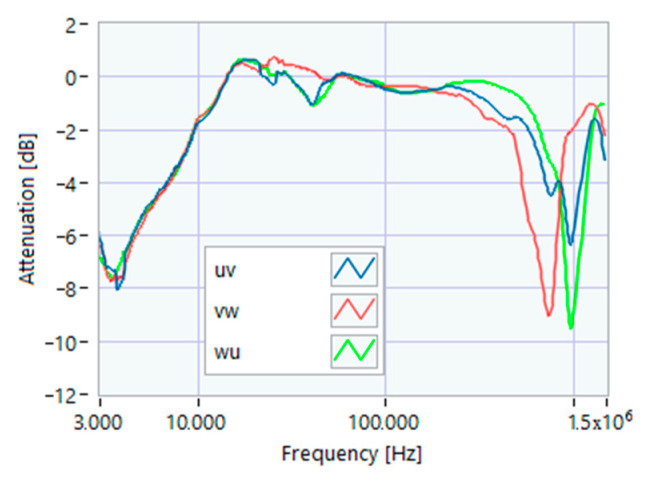
SFRA online TF of the supply system only, measured on line-to-line terminals.

**Figure 4:**
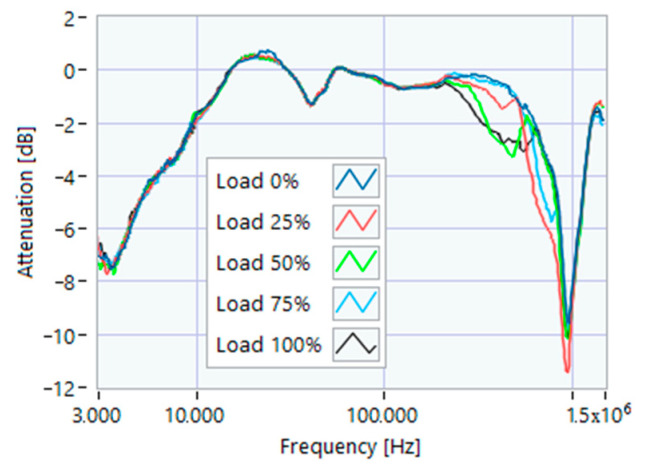
SFRA online motor 1 at various load percentages after 1 h of full load service.

To this aim, we first applied the SFRA to the supply system with the motor circuit breaker open (Figure 3). The three TFs were different, so it was possible to limit further analysis to the terminals showing the most regular shape. In this study, the line-to-line terminals were selected for further acquisitions because they only had one resonance peak in the range of 800–1500 kHz, and they were at a higher frequency value compared to the others. Figure 4 shows the SFRA TFs measured on motor 1 at various load percentages after 1 h of full load service. Since no effect was appreciable below 100 kHz, it was decided to limit the measurement band in the range of 100 kHz–1.5 MHz. For each motor, five TFs were acquired for mechanical loads varying from 100% to 0%, always after one hour of operation at full load.

**Figure 5:**
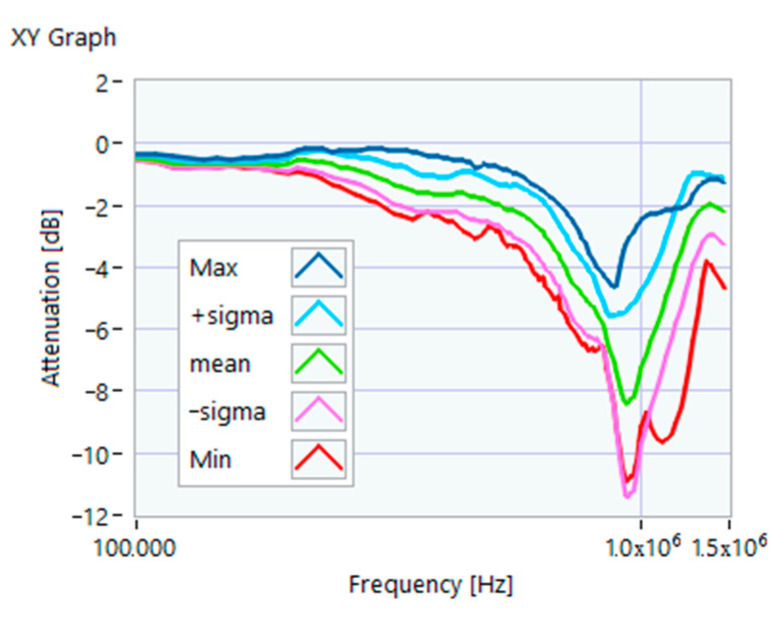
Reference TF processed at 0% load.

**Figure 6:**
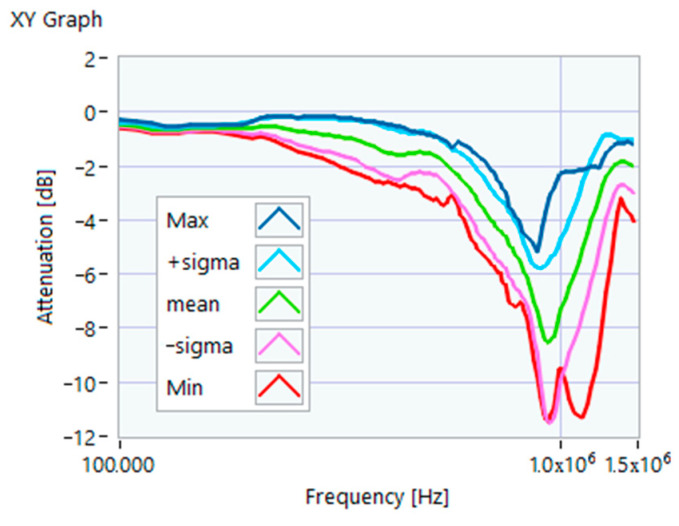
Reference TF processed at 25% load.

**Figure 7:**
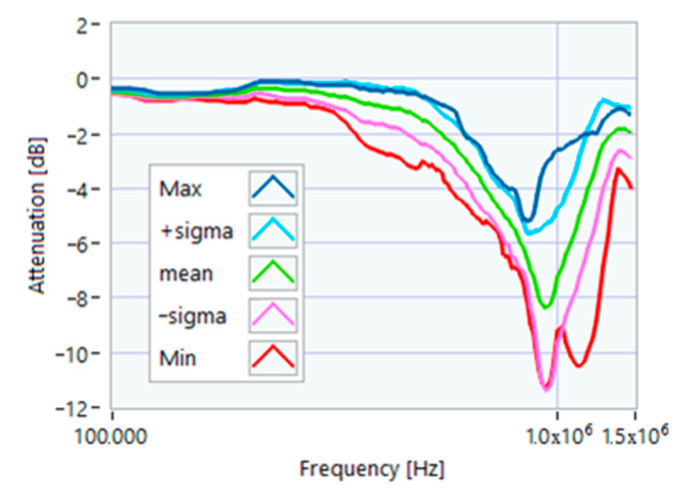
Reference TF processed at 50% load.

**Figure 8:**
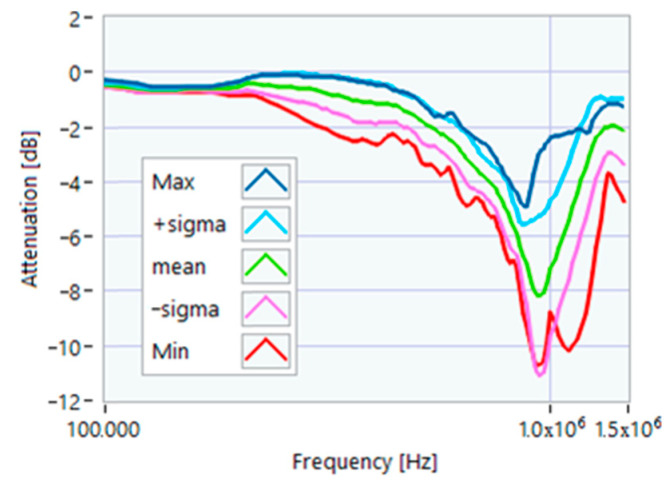
Reference TF processed at 75% load.

**Figure 9:**
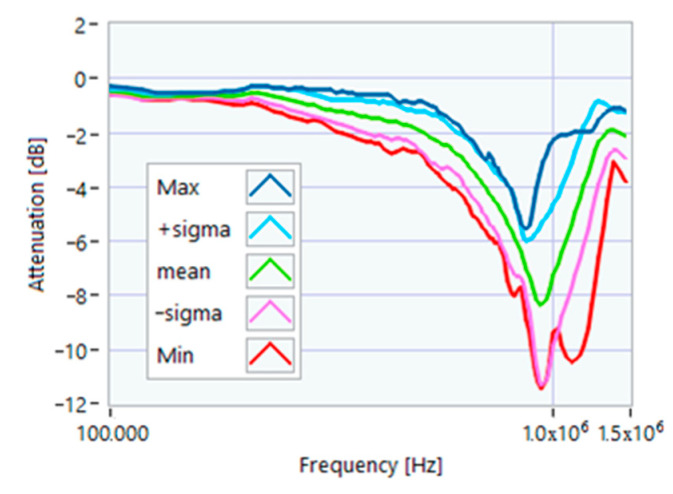
Reference TF processed at 100% load.

**Figure 10:**
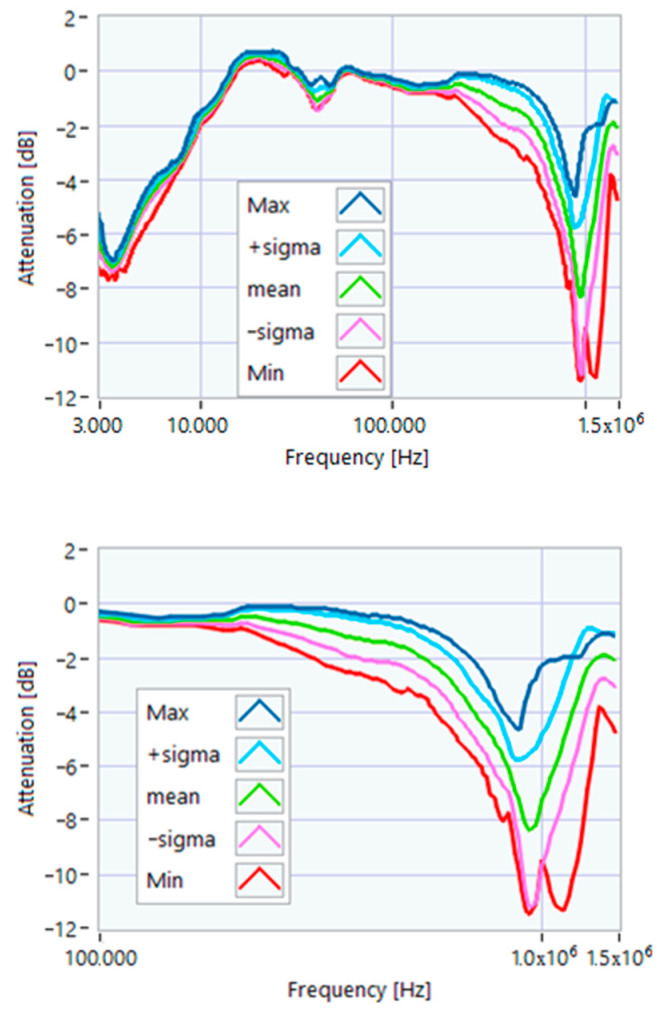
Overall reference TFs processed with the whole dataset.

The definition of the reference curves was first performed by considering the TFs corresponding to the same working point, obtaining the envelope TFs shown in Figure 5, Figure 6, Figure 7, Figure 8 and Figure 9. Each figure shows: (1) the TF of the envelope of the maximum values (indicated as Max); (2) the TF of the envelope of the minimum values (Min); (3) the average TF (mean); (4) the TF obtained as the average plus the standard deviation for each frequency value (+sigma); (5) the TF obtained as the mean minus the standard deviation (-sigma). This choice allowed for identifying the different degrees of deviation in a subsequent experimental TF. For example, the standard deviation allowed for defining a region that included 68% of all the data points around the mean TF (““+sigma”” and ““-sigma”” TFs in Figure 5, Figure 6, Figure 7, Figure 8, Figure 9 and Figure 10 and following). In Figure 10, the total reference TFs, obtained with the dataset of 50 acquisitions, are reported in the range of 3 kHz–1.500 MHz and the subrange of interest from 100 kHz to 1.500 MHz. Below 100 kHz, the TFs remained almost similar, confirming that the effect of the presence of the motor was not relevant in this band.

## 4. The Proposed Analysis of Experimental Data

The evaluation of the data obtained with the SFRA technique is usually carried out with a qualitative approach, displaying the reference TFs compared to the current TF with the support of numerical indicators, which can be of a statistical type. In line with this modality, a first check was conducted to investigate whether the TF was within the TF envelope, defined by the maximum and minimum TFs elaborated in Section 3. The percentages of the frequency values for which the TF is above the maximum or below the minimum can be indicative of a failure still in the initial phase, even if no other index confirms appreciable changes. This check can be carried out on the whole TF or on parts of it to emphasize local effects.

As a second test, correlation methods were adopted and applied to the entire extension of the TF or only to the bands considered significant. In more detail, in this study, two correlation indices were considered. The first index was Pearson’s correlation *ρ*:

(1) $$\rho =\frac{n\sum^{\;}x_{i}y_{i}-\sum^{\;}x_{i}\sum^{\;}y_{i}}{\sqrt{n\sum^{\;}x_{i}^{2}-{\left(\sum^{\;}x_{i}\right)}^{2}}\sqrt{n\sum^{\;}y_{i}^{2}-{\left(\sum^{\;}y_{i}\right)}^{2}}}$$

where *n* is the number of TF values, xi and yi are the values of the X and Y TFs, respectively (for the ith sample). Pearson’s correlation is the most commonly used in the SFRA context; it measures the linear correlation between two TFs.

The second was the Spearman rank correlation coefficient *r*, which is helpful if the TFs being compared make the Pearson correlation coefficient undesirable or misleading. The Spearman correlation measures the strength and direction of the monotone association between the two TFs, according to the equation:

(2) $$r=1-\frac{6\sum^{\;}d_{i}^{2}}{n\left(n^{2}-1\right)}$$

where *d_i_* is the difference in the paired ranks and *n* is the number of values. Kendall’s *τ* and other correlation coefficients were disregarded as they could not provide further information. The coefficient evaluation procedure was based on the qualitative observation that the motor effect was insignificant for frequencies lower than 100 kHz. Therefore, it was possible to carry out correlation tests with *ρ* and *r* in the band from 3 kHz to 100 kHz to mainly observe variations in the supply network impedance in the motor connection node. The correlation test was based on the following observation: in the range from 100 kHz to 1.5 MHz, the motor under examination showed more resonance peaks than the average TF of the motors. However, in the specific case of the motor in question, there was only one peak at about 950 kHz. To use the Spearman r correctly, the flat, increasing, and decreasing sections of the TF must be isolated. Therefore, an algorithm was implemented to divide the TF under test into flat sections and calculate the coefficients *ρ* and *r* in the identified sections. The appearance of additional peaks or the shift of the primary resonance peak was simultaneously detected by *ρ* and ρ. Since five reference TFs were available, as shown in Figure 5, Figure 6, Figure 7, Figure 8 and Figure 9, the solution of calculating the *ρ* and *r* for all TFs was adopted to identify the worst similarity conditions. The deformations that should have had the most significant effect on the correlation indices were the slope changes, the emergence of new secondary peaks, and the displacement of the main peak. The latter phenomenon may be related to the variation in the parasitic inductive and capacitive parameters at higher frequencies, which could be monitored to reveal the most invasive damage.

## 5. Characterization of the Motors with Slight Damages

In some of the motors previously tested, some minor damage was produced, which still allowed the motors to operate without showing apparent malfunctions. They were selected by considering both slight electrical and mechanical issues that can happen during assembly or maintenance operations or due to overloading or production defects. They can be considered the most probable for low-cost industrial induction motors. The main purpose was to verify that they induced changes to the TFs that were detectable through the online SFRA. A total of nine cases of damage occurred, one for each motor, as described below. The motor of the group analyzed with the TF at full load closest to the average TF was excluded to be used as a reference for further tests. The studied frequency range was from 100 kHz to 1.500 MHz to minimize the effects involved by the impedance variations of the power supply network, which were not predictable during normal operation. All TFs in the figures were acquired under the same working conditions after 1 h of service at full load.

### 5.1. Increased Resistance of a Winding

**Figure 11:**
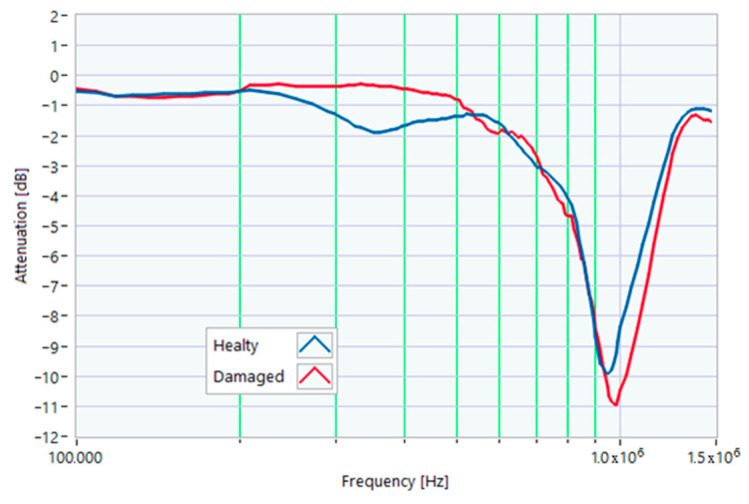
Comparison between TFs of the same motor in healthy conditions and with a 1 Ω resistance-in-series on a phase conductor.

**Figure 12:**
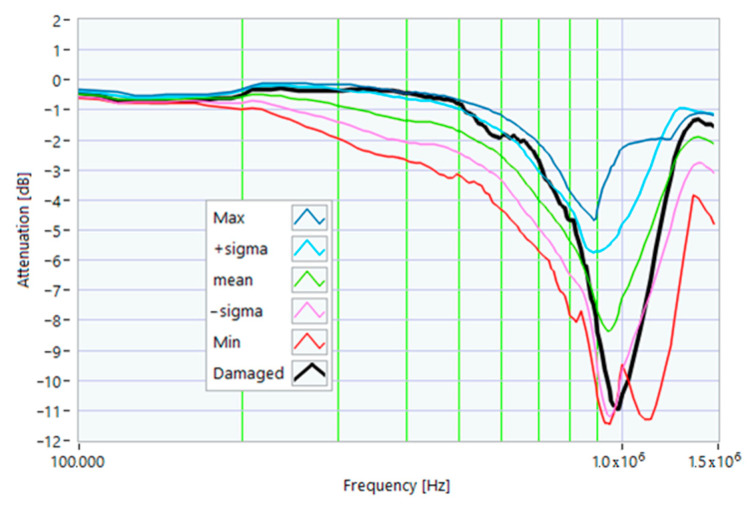
Comparison of the TF of a motor with a 1 Ω resistance-in-series on a phase conductor with the reference TFs on the whole bandwidth.

The first damage was obtained by inserting a power resistor of 1 Ω resistance-in-series with one of the line conductors. According to the rated values of the motor, the average phase impedance was about 38 Ω at full load. Therefore, the inserted resistance involved a variation in one of the windings of 2.6% of impedance. This condition can be similar to an incorrect terminal block screw tightening, which increases the tightening resistance or a section reduction of one of the terminal block conductors. A comparison between the TFs of the same motor before and after the resistance variation is shown in Figure 11. There was an appreciable difference in the frequency range from 200 kHz to 500 kHz and slight deformations in the range from 500 kHz to 990 kHz. The main peak shifted from 950 kHz to 982 kHz. In Figure 12, the TF of the damaged motor seems to be inside the limits of the max and min envelope TFs. The same TF of the damaged motor shown in Figure 11 was analyzed, as discussed in Section 4. No TF values exceeded the max TF envelope in the frequency range from 100 kHz to 1.500 MHz, while 1.81% were under the min TF envelope. Therefore, the main peak at 982 kHz can be assumed as an initial warning condition, as confirmed by the correlation analysis.

Table 1 presents the correlation coefficients processed from 100 kHz to 982 kHz. Table 2 shows the same coefficients for the 982 kHz to 1.5 MHz range. Again, all the processed correlation indexes were above 0.8, with the higher values for linear correlations (ρ) with the reference “–sigma” TF in the range from 100 kHz to 982 kHz and the mean TF in the range from 982 kHz to 1.500 MHz.

### 5.2. Leakage Current under the Residual Current Circuit Breaker-Tripping Threshold

**Figure 13:**
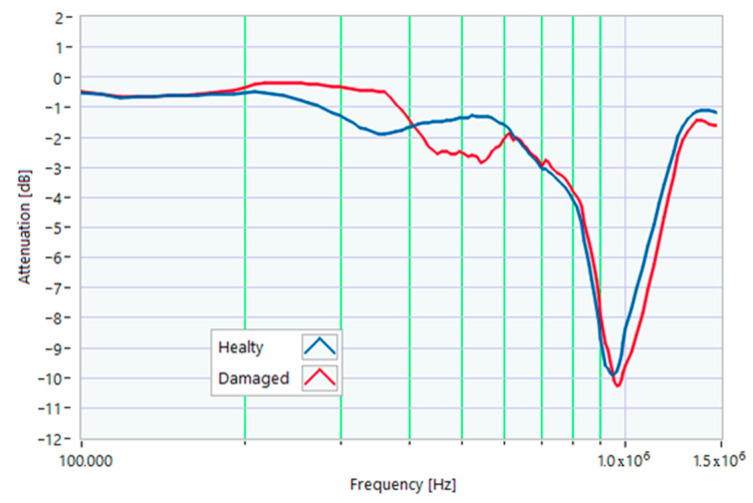
Comparison between TFs of the same motor in healthy conditions and with a 25 kΩ resistance between one terminal and the ground.

The second damage was obtained by inserting a 25 kΩ resistor between one winding terminal and the ground to simulate an initial insulation fault condition, with a leakage current lower than the trip current of a standard 30 mA residual current circuit breaker. Similarly, to the previous case, the most noticeable difference was in the range from 200 kHz to 950 kHz. In addition, there were slight variations in other pieces of the TF (Figure 13).

**Figure 14:**
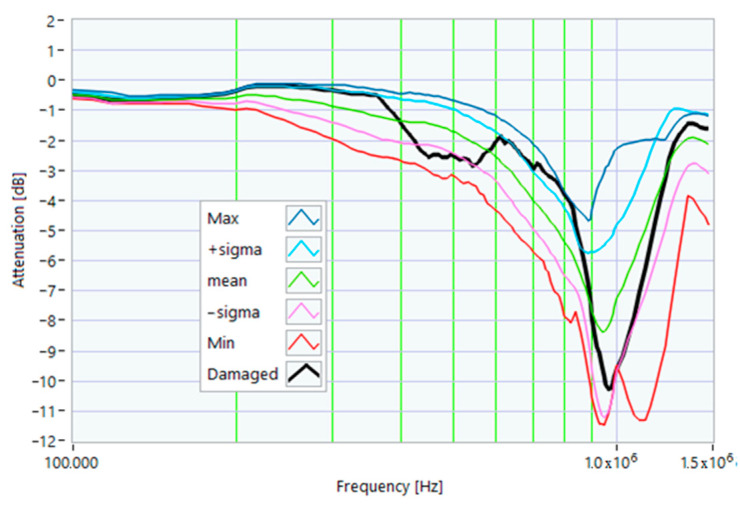
Comparison of the TF of a motor with a 25 kΩ resistance between one terminal and the ground with the reference TFs.

The minor deformations of the TF due to the leakage resistance can be related to the small residual current which can be present even in normal working conditions. Interestingly, the shape of the TF was modified but was essentially within the range bounded by the maximum and minimum envelope TFs (Figure 14).

From the TF analysis of the damaged motor in Figure 13, in the frequency range from 100 kHz to 1.5 MHz, 0% of the values exceeded the maximum TF envelope, while 0.45% were below the TF envelope minimum. Therefore, this was a very initial warning condition. The main peak was at a frequency of 973 kHz. The correlation coefficients processed are shown in Table 3 for the range from 100 kHz to 973 kHz, and in Table 4 for the range from 973 kHz to 1.5 MHz. The higher correlation in the first range was linear (ρ) with the reference “– sigma” TF but with a lower value (0.9591) in comparison to the previous case because of the slope inversion in the range from 200 kHz to 600 kHz, as shown in Figure 13. On the other hand, in the band from 973 kHz to 1.500 kHz, there were good correlations for almost all reference TFs.

### 5.3. Rotor Misalignment

**Figure 15:**
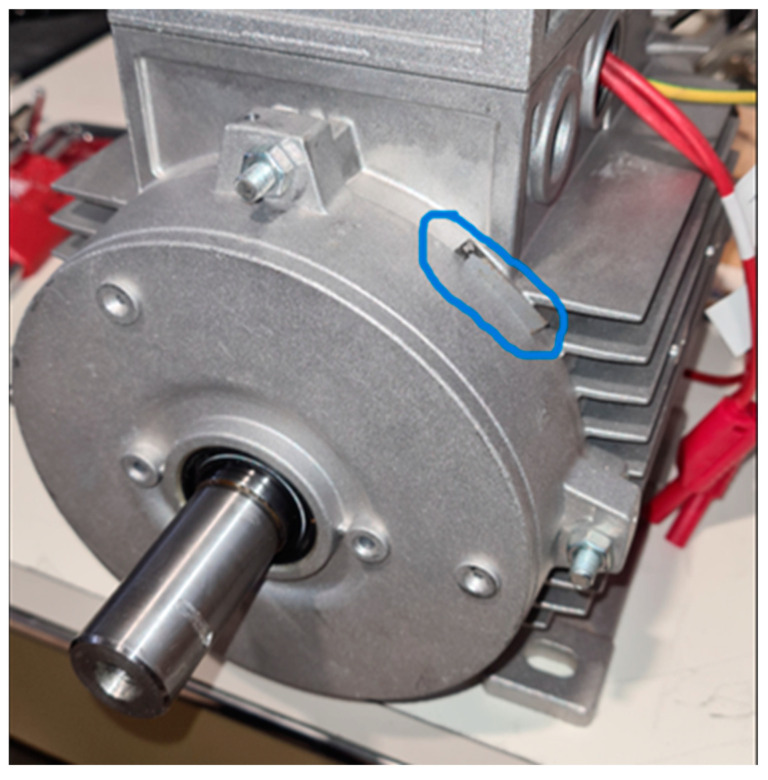
Induction motor with slight rotor misalignment.

**Figure 16:**
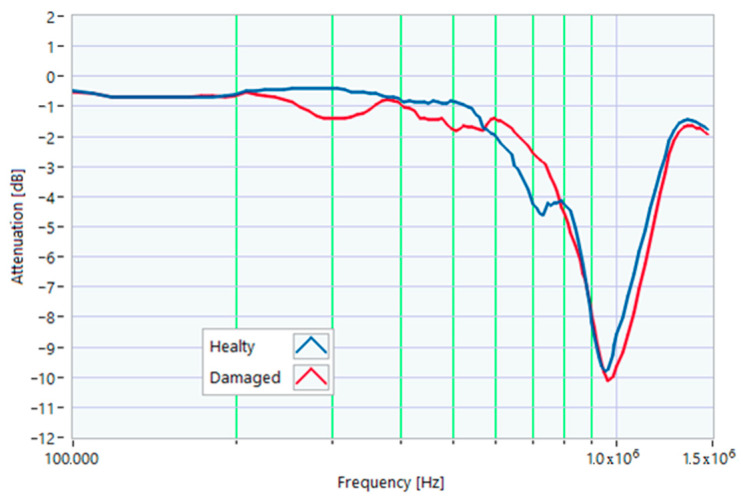
Comparison between TFs of the same motor in healthy conditions and with slight rotor misalignment.

**Figure 17:**
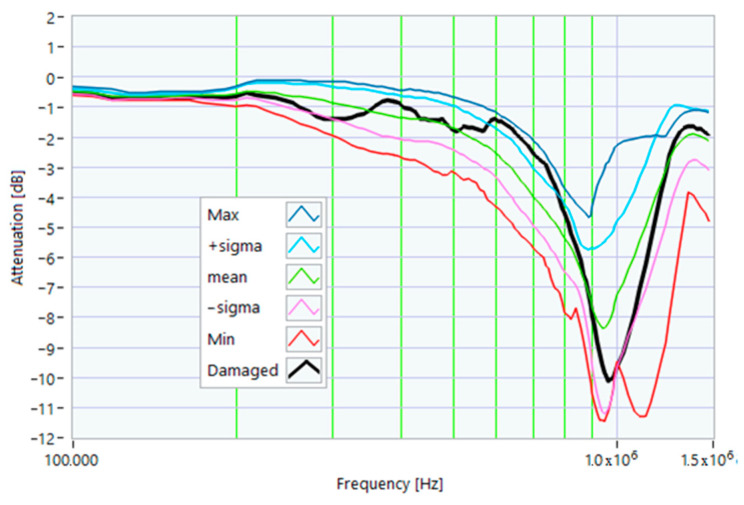
Comparison between the TF of the motor with slight rotor misalignment and the reference TFs.

Another anomalous condition is due to the incomplete tightening of the motor shield screws, resulting in slight rotor misalignments. This condition was obtained by inserting a 0.3 mm thick metal sheet between the shield and the frame in the position shown in Figure 15. Also in this case, the variations in the TF were more evident in the range from 200 kHz to 950 kHz. Minor variations in the TF were within the maximum and minimum envelope of TFs. This type of slight misalignment is very common, and therefore, the TF obtained with a slight variation is compatible with a typical working condition. However, this situation still needs to be monitored to avoid growing misalignments due to vibrations linked to torque undulations (Figure 16 and Figure 17). From the TF analysis of the damaged motor in Figure 15, in the frequency range from 100 kHz to 1.5 MHz, 0% of the values exceeded the maximum TF envelope, while 0.44% were below the TF envelope minimum. Therefore, even this anomaly can be assumed as a very early warning condition, possibly confirmed by the correlation analysis. The main peak was at a frequency of 964 kHz. The correlation coefficients processed are in Table 5 for the range from 100 kHz to 964 kHz, and in Table 6 for the range from 964 kHz to 1.5 MHz. The slight TF deformation in Figure 15 led to good correlation results in both frequency ranges.

### 5.4. Overheating

**Figure 18:**
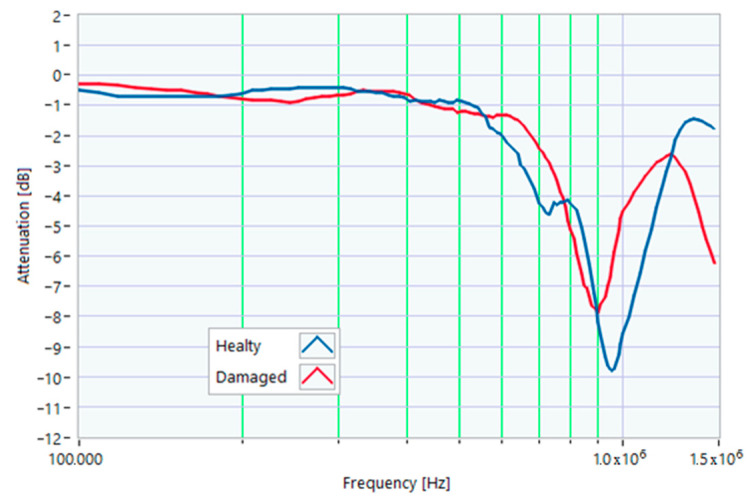
Comparison between TFs of the same motor in healthy conditions and after overheating.

**Figure 19:**
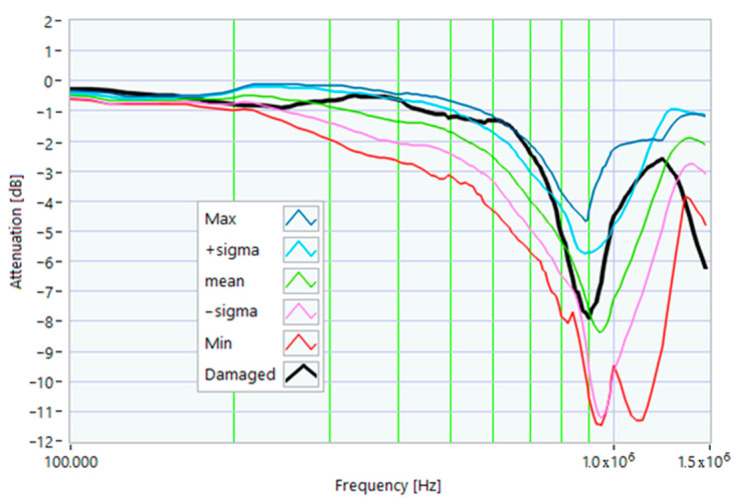
Comparison of the TF of a motor after overheating with the reference TFs.

A significant cause of failure is the motor overheating due to inefficient cooling or overloads for long periods. Taking into account the insulation class of the tested motor, which is 140 °C, we placed the motor in a climatic chamber at 150 °C for 5 h to simulate overheating. The motor was tested after 24 h, allowing it to reach thermal equilibrium with the environment. After this procedure, the TF showed significant differences for frequencies above 500 kHz, as shown in Figure 18. The comparison between the TF after overheating and the envelope TFs detected under normal conditions shows that the motor appeared to operate under regular operating conditions, within the envelope of the max and min TFs (Figure 19).

Based on the analysis of the damaged motor TF shown in Figure 19 in the frequency range from 100 kHz to 1.5 MHz, it appears that 16.36% of the TF values exceeded the maximum TF envelope, while 2.72% were below the minimum TF envelope. This is, therefore, a medium alert condition, which had to be confirmed by the correlation analysis. The main peak was at a frequency of 901 kHz.

The correlation coefficients processed are shown in Table 7 for the range from 100 kHz to 901 kHz, and in Table 8 for the range from 901 kHz to 1.5 MHz. The shift in the main peak resulted in low correlation values for both ρ and r, shown in Table 8, thus confirming the severity of the damage that required predictive maintenance.

### 5.5. Stator Fall

**Figure 20:**
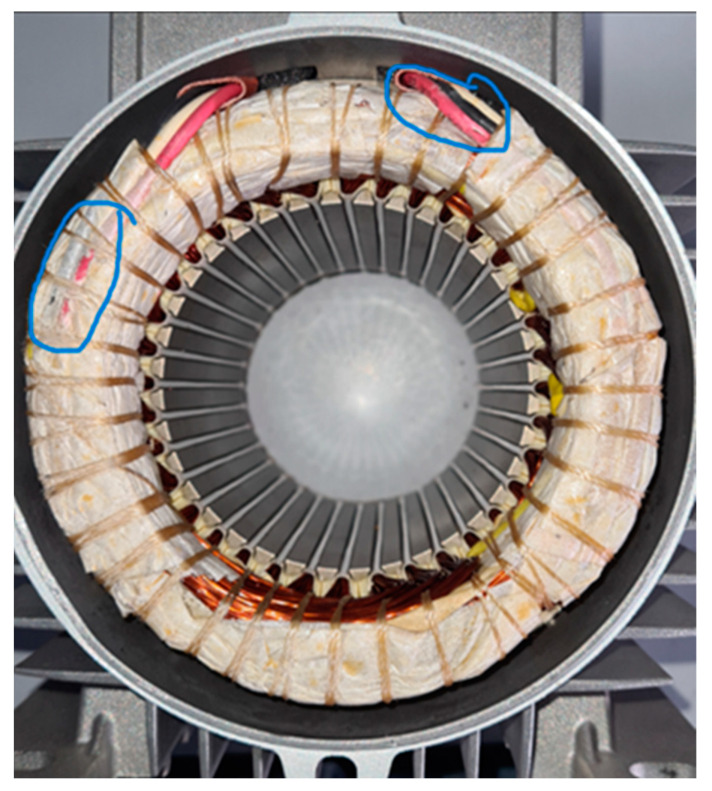
The stator of an induction motor after a fall from a height of 1 m.

Accidents can occur during and after the manufacture of a motor. For example, the stator could be subjected to shocks or falls, not seriously damaging the winding insulation. This type of event can be considered non-critical to the behavior of the motor. However, it can lead to insulation failures during service if the coil deformations are significant. The stator of one of the motors was dropped on the floor from a height of 1 m. The damage to the winding head is highlighted in Figure 20.

**Figure 21:**
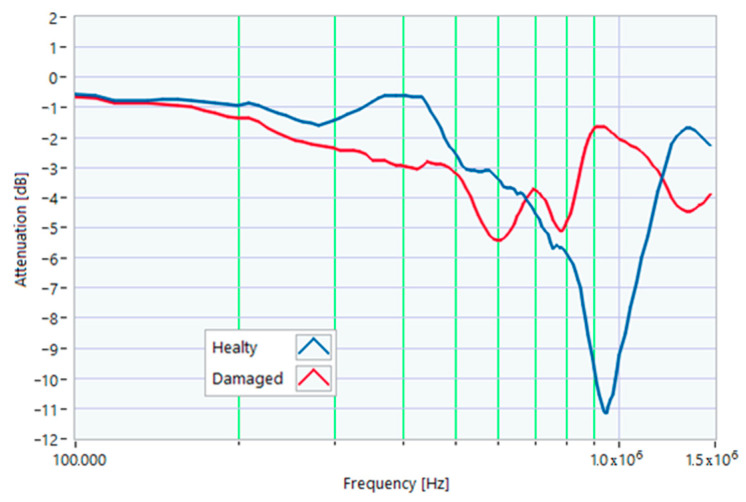
Comparison between TFs of the same motor in healthy condition and a stator fell from a height of 1 m.

**Figure 22:**
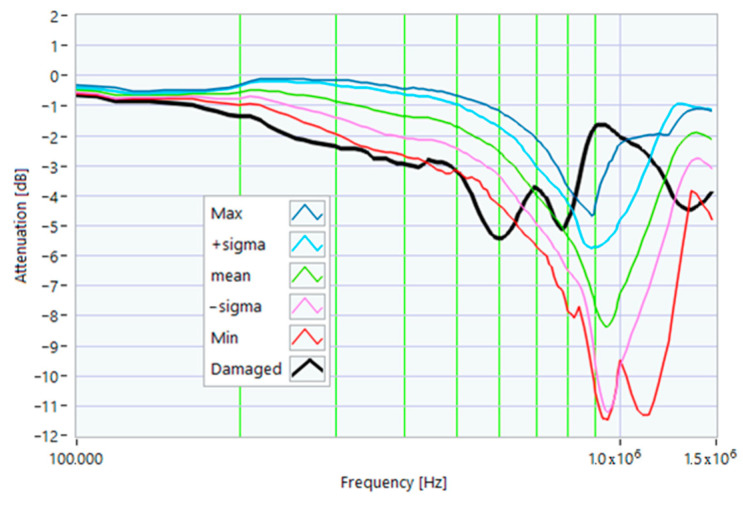
Comparison of the TF of a motor after the stator fell from a height of 1 m with the reference TFs.

The comparison of TFs in Figure 21 shows significant differences for almost all the frequencies. A new peak occurred at 71.65 kHz, and the TF had a very different shape at higher frequencies, with a new peak at about 600 kHz. The main peak was attenuated and shifted from 950 kHz to 760 kHz. The last peak shifted from 1.3 MHz to 930 kHz. The comparison with the reference TFs in Figure 22 shows that the TF was outside the min and max TFs in almost the whole frequency range considered. Despite this, the motor appeared to be in normal condition during the testing. Changes in the TF could be linked to deformations induced by the fall, affecting the winding turns’ parasitic capacitance. From the analysis of the damaged motor TF in Figure 20, in the frequency range from 100 kHz to 1.5 MHz, it appears that 9.54% of the TF values exceeded the max TF envelope, while 70.91% were under the min TF envelope. Therefore, this is a high-warning condition, confirmed by the correlation analysis. The main peak was at a frequency of 604 kHz, but the TF shape was very deformed. The correlation coefficients processed are in Table 9 for the range from 100 kHz to 604 kHz, and in Table 10 for the range from 604 kHz to 1.5 MHz. Although the correlation values in the range from 100 kHz to 604 kHz were good, the negative values in the range from 604 kHz to 1.500 MHz reveal the intense TF deformation, confirming the severity of the damage.

### 5.6. Shorting Ring Damage

**Figure 23:**
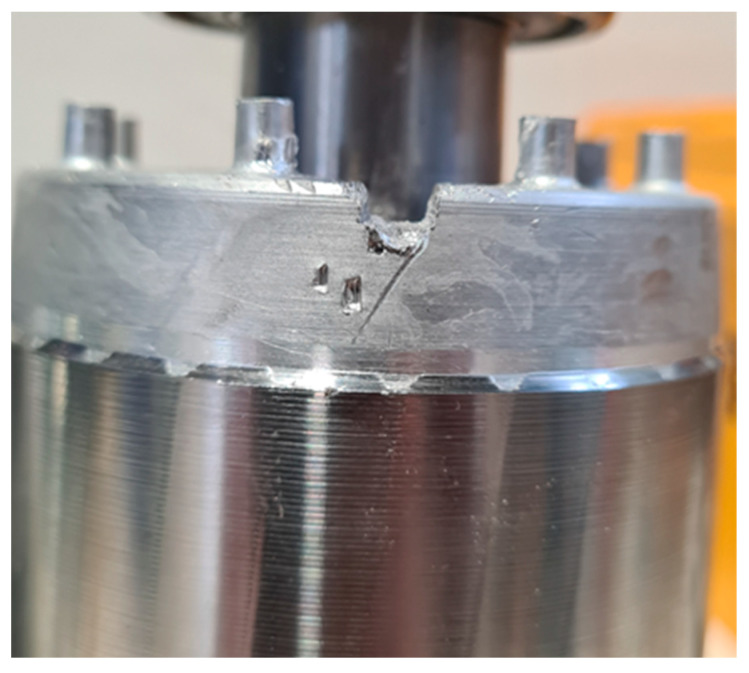
Shorting ring damage.

**Figure 24:**
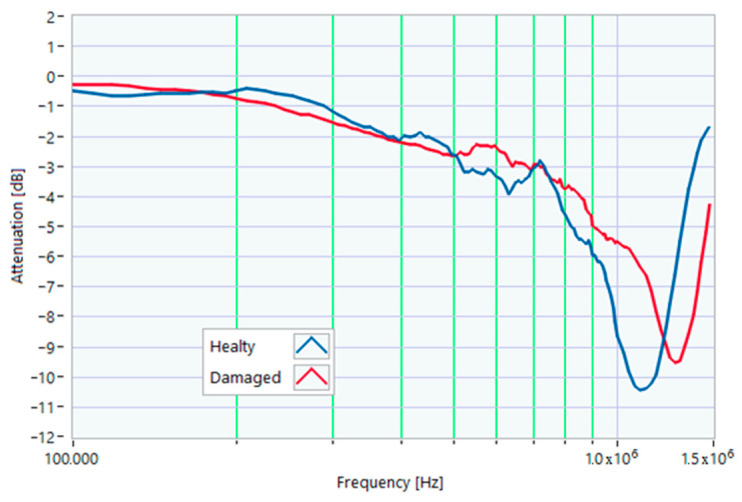
Comparison between TFs of the same motor in healthy conditions and with shorting ring damage.

**Figure 25:**
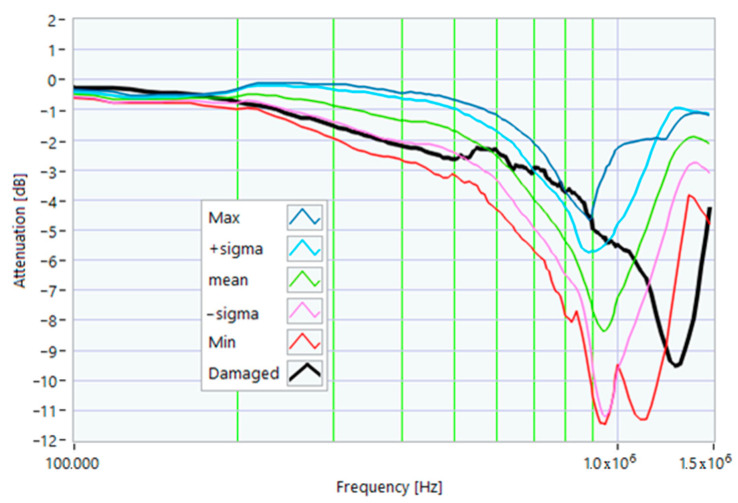
Comparison between the TF of a motor with shorting ring damage and the reference TFs.

The shorting rotor ring of a squirrel cage induction motor is usually made of diecast aluminum. In some cases, a clique or a casting defect can affect the ring, affecting the ring’s resistance. One of the rotors of the motors batch was damaged by removing a part of the ring using a cutter, as illustrated in Figure 23. The TFs in Figure 24 differed significantly for frequencies higher than 400 kHz. A shift in the main peak from 1.1 MHz to 1.275 MHz can be seen. The damaged rotor TF is partially outside the max and min TF envelope, as shown in Figure 25.

Analyzing the TF of the damaged motor (Figure 25) in the frequency range from 100 kHz to 1.5 MHz, it appears that 35.0% of the TF values exceeded the TF max envelope, while 4.54% were below the envelope TF min. This is, therefore, a condition of average alert, also confirmed by the correlation analysis. The main peak was at a frequency of 1.275 MHz.

The correlation coefficients processed are shown in Table 11 for the range from 100 kHz to 1.275 MHz, and in Table 12 for the range from 1.275 MHz to 1.500 MHz. Some of the correlation results from 100 kHz to 1.275 MHz (Table 11) are below 0.65. Two negative values in Table 12 reveal the substantial variation in the TF induced by this damage.

### 5.7. Rotor Bar Damage

**Figure 26:**
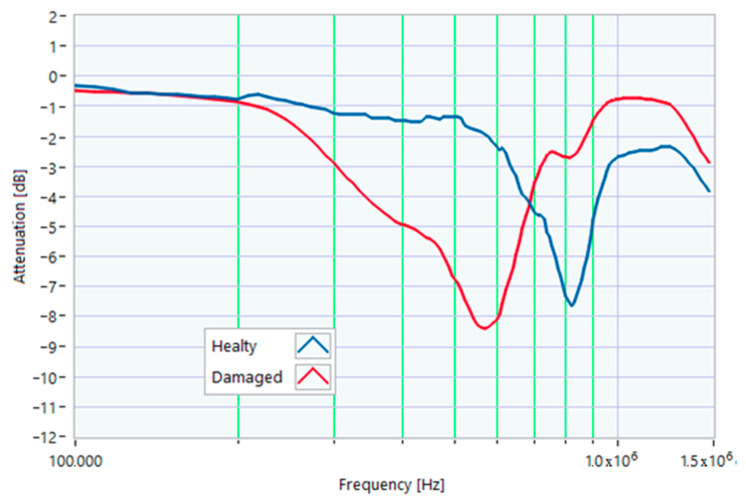
Comparison between TFs of the same motor in healthy conditions and with rotor bar damage.

**Figure 27:**
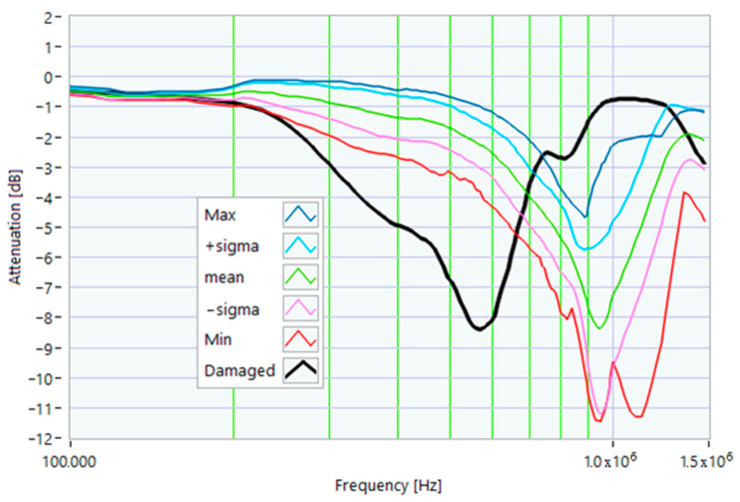
Comparison of the TF of a motor with shorting ring damage with the reference TFs.

In this case, a small hole (1 mm in diameter) was drilled in one of the rotor slots to damage one of the aluminum bars of the rotor. This type of damage is similar to that due to casting defects, such as voids or cracks. The TFs in Figure 26 show the effect of this damage, with a significant deformation in the range from 200 kHz to 1.500 MHz. The main peak shifted from 820 kHz to 570 kHz. The TF of the damaged motor was below the min envelope TF in an extensive frequency range, as depicted in Figure 27. This behavior could be helpful in data processing for identifying the incoming fault.

From the analysis of the damaged motor TF of Figure 27, in the frequency range from 100 kHz to 1.5 MHz, it appears that 18.0% of the TF values exceeded the max TF envelope, while 61.36% were under the min TF envelope. Therefore, the correlation analysis confirms that this is a critical warning condition. The main peak was at a frequency of 568 kHz.

The correlation coefficients processed are shown in Table 13 for the range from 100 kHz to 568 kHz, and in Table 14 for the range from 568 kHz to 1.500 MHz. The correlation results in Table 14, which are all negative because of the shift in the main peak, confirm the severity of the damage.

### 5.8. Rotor Group Fall

**Figure 28:**
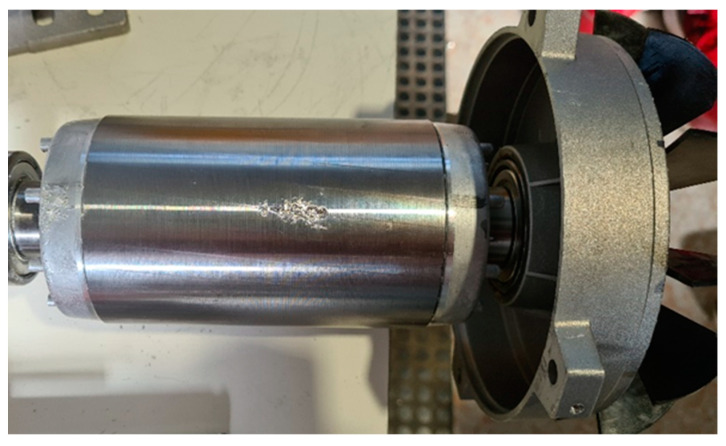
Rotor group fall.

**Figure 29:**
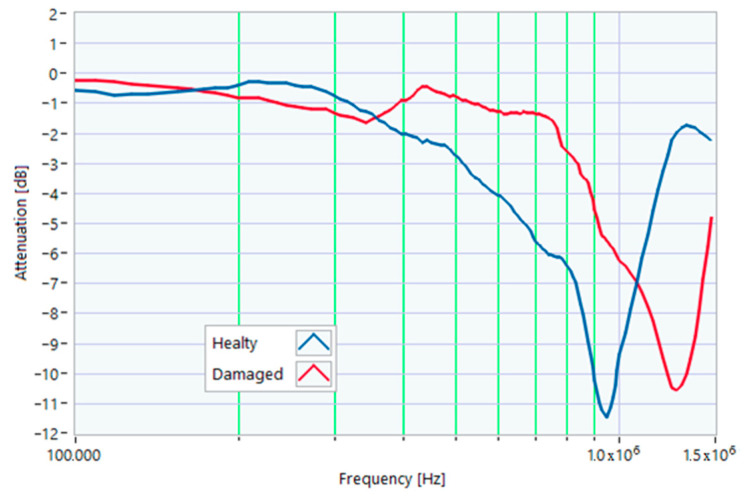
Comparison between TFs of the same motor in healthy conditions and after the rotor group fell from a height of 1 m.

In this test, the rotor–fan–shield assembly was dropped to the ground from a height of 1 m; the impact deformed the fan and the shield, as shown in the photo of Figure 28. This type of damage can also occur during assembly or maintenance. Significant effects were expected due to possible eccentricity related to the resulting deformations. An effect of the drop was a shift in the main peak, shown in Figure 29, from 950 kHz to 1.275 MHz. The TF obtained was partially outside the minimum and maximum range, as in the previous cases.

**Figure 30:**
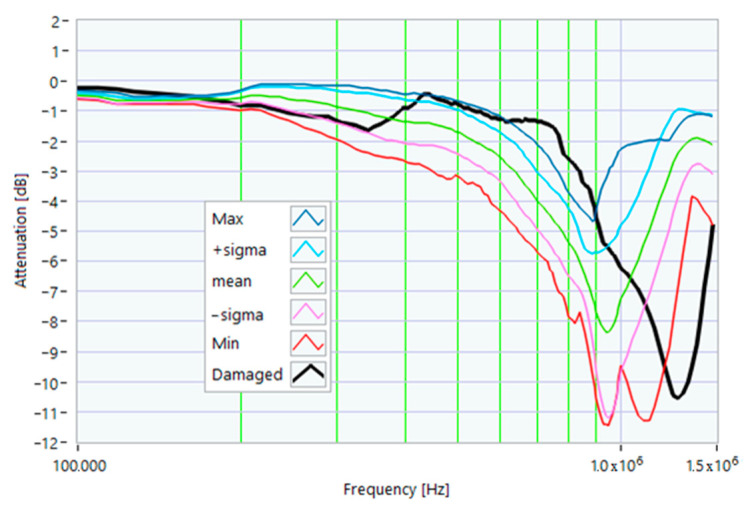
Comparison of the TF of a motor after the rotor group fell from a height of 1 m with the reference TFs.

From the analysis of the damaged motor TF in Figure 30, in the frequency range from 100 kHz to 1.5 MHz, it appears that 48.63% of the TF values exceeded the max TF envelope, while 4.54% were under the min TF envelope. Therefore, the correlation analysis confirms the critical warning condition. The main peak was at a frequency of 1.275 kHz. The correlation coefficients processed are shown in Table 15, for the range from 100 kHz to 1.275 MHz, and in Table 16, for the range from 1.275 MHz to 1.500 MHz. The correlation results tabulated in Table 15 are generally low, while two negative values in Table 16 confirm the severity of the damage.

### 5.9. Short Circuit between Winding Turns

**Figure 31:**
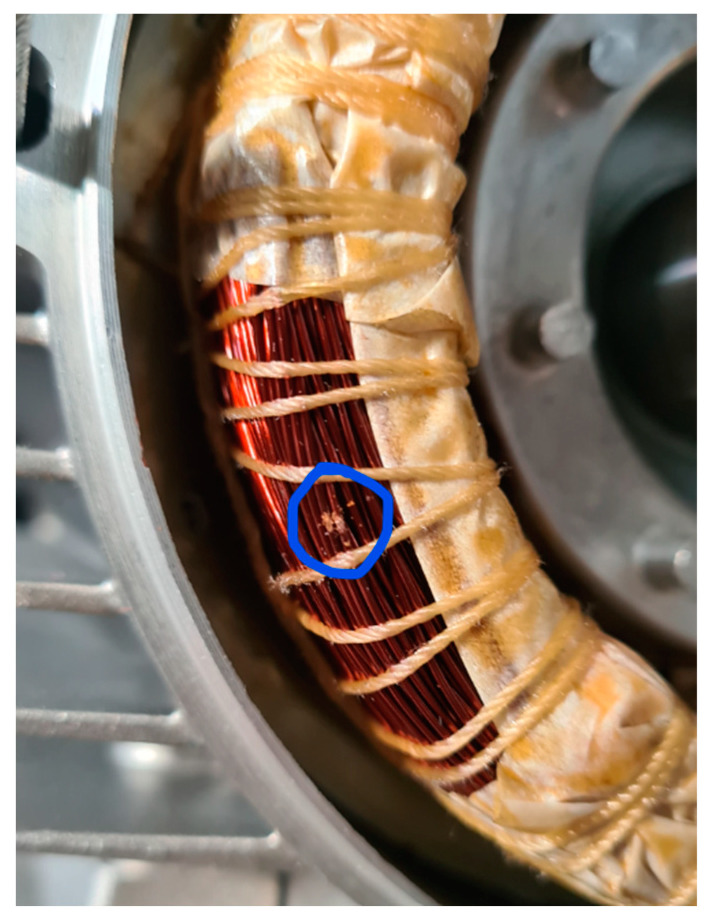
Insulating enamel removed.

**Figure 32:**
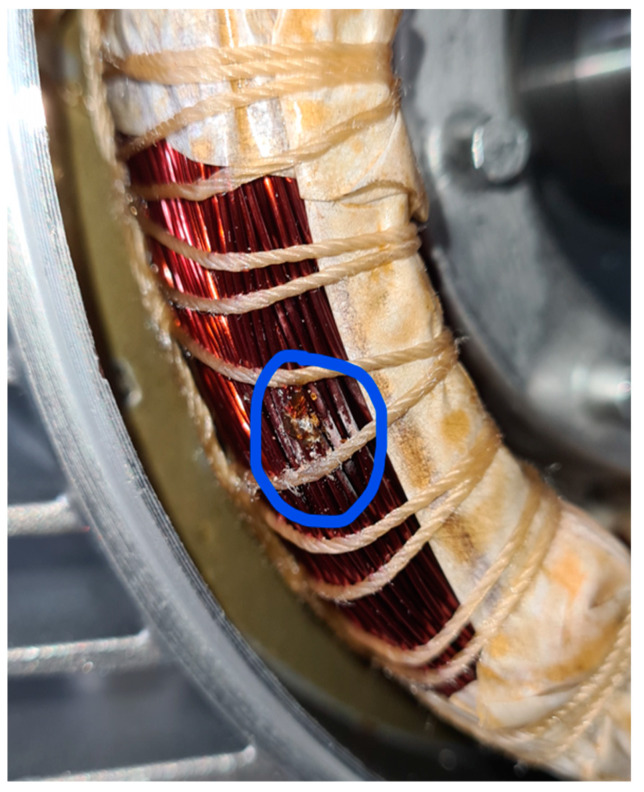
Welded winding turns.

The last analyzed damage was a shortcut between two winding turns, obtained by scraping the insulating enamel and soldering the two copper conductors (Figure 31 and Figure 32). This defect, which can manifest due to errors in the glazing or the winding assembly process, can easily lead to dangerous failures.

**Figure 33:**
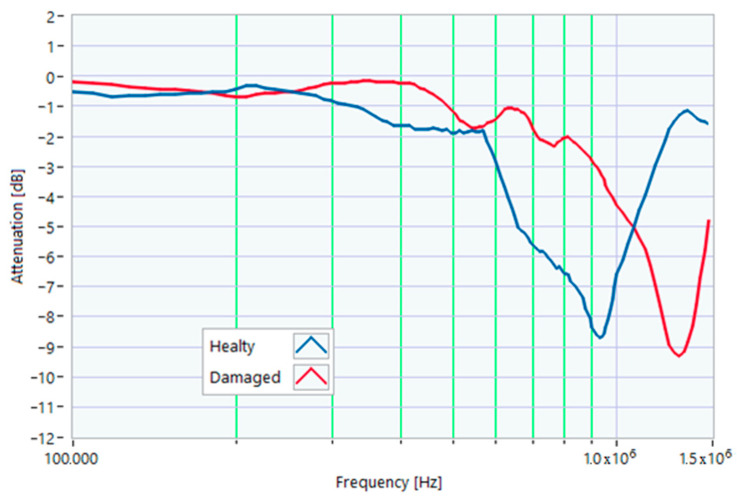
Comparison between TFs of the same motor in healthy conditions and with a shortcut between 2 winding turns.

**Figure 34:**
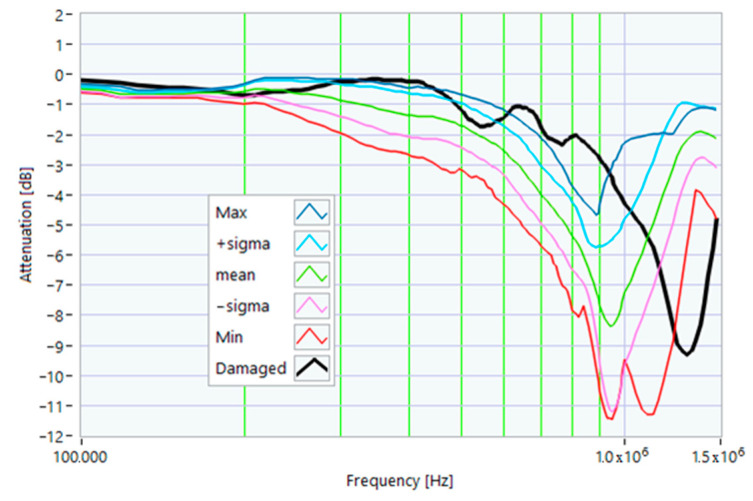
Comparison between the TF of a motor with a shortcut between 2 winding turns and the reference TFs.

From the analysis of the damaged motor TF in Figure 33 and Figure 34, in the frequency range from 100 kHz to 1.5 MHz, it appears that 59.54% of the TF values exceeded the max TF envelope, while 4.09% were under the min TF envelope. Therefore, this is another critical warning condition, confirmed by the correlation analysis. The main peak was at a frequency of 1.300 kHz.

The correlation coefficients processed are shown in Table 17 for the range from 100 kHz to 1.300 MHz, and in Table 18 for the range from 1.300 MHz to 1.500 MHz. The linear correlation results (ρ) shown in Table 17 are low, and the negative values shown in Table 18 confirm this severity of damage.

## 6. Conclusions

In this article, a batch of asynchronous motors was characterized in healthy and damaged conditions using the SFRA online technique. The goal was to identify a predictive diagnostic procedure for grid-powered motors capable of recognizing possible deviations from regular operation characteristics before catastrophic failures occur. With the aid of correlation indices determined in specific TF sub-bands, it is proposed to support the qualitative analysis of TFs with data to be included in the predictive diagnostic algorithm.

The correlation analysis can give quantitative information about the TFs’ shape variations, strongly depending on the features and sizes of the motor set. After an extended measurement campaign on batches of motors, it will be possible to identify reference values for correlation indices to be adopted with a strictly quantitative approach.

The characterization of the motors under healthy conditions led to the elaboration of some reference TFs, with which the TFs measured on the motors after the occurrence of specific damages were compared. Nine typologies of typical damage were considered as case studies: a small increment of one winding resistance, a small leakage current to the ground, a rotor misalignment, a motor overheating, a stator and rotor group falling, damage to the shorting ring and one of the rotor bars, and a short circuit between two turns of the same winding.

Since the analyzed damage was fully compatible with apparent good functioning of the motor, it was challenging to predict the start of the faults with the measurement of the typical mechanical and electrical quantities. Nevertheless, from the obtained results, the online SFRA technique appears to be suitable for carrying out predictive monitoring, thanks to its sensitivity even to reduced variations in the measured parameters, and above all, through the analysis of correlations.

The 1.5 kW motor size was chosen because it is among the most widespread in the industrial field, but the results obtained are generally applicable to motors of a larger size. The larger the motor size, the higher its impact on the resulting impedance in the grid. With larger motors, TF variations due to slight damages are expected to be detected more easily.

The cost and the mission of larger-sized motors can justify the adoption of the proposed technique if predictive maintenance is one of the goals to be achieved. It could be of interest for induction motors adopted for the cooling of critical power system components or to prevent power quality phenomena involved in motor faults.

In the future, a new online SFRA system will be implemented to have three channels of simultaneous signal injection. Thus, it will be possible to acquire TFs on all three windings by automatically changing the input terminals at which the input signal is acquired.

Finally, more tests will be performed considering wound rotor induction motors.

## Figures and Tables

**Table 1:** Correlation factors in the range from 100 kHz to 982 kHz for a motor with a 1 Ω resistance-in-series on a phase conductor.

Reference TF	Pearson’s ρ	Spearman r
Max	0.8618	0.9671
+ sigma	0.9436	0.9692
mean	0.9719	0.9268
- sigma	0.9771	0.9054
Min	0.9557	0.8999

**Table 2:** Correlation factors in the range from 982 kHz to 1.500 MHz for a motor with a 1 Ω resistance-in-series on a phase conductor.

Reference TF	Pearson’s ρ	Spearman r
Max	0.9082	0.9844
+ sigma	0.9906	0.8857
mean	0.9992	0.9974
- sigma	0.9949	0.9987
Min	0.8110	0.8221

**Table 3:** Correlation factors in the range from 100 kHz to 973 kHz for a motor with a 1 Ω resistance-in-series on a phase conductor.

Reference TF	Pearson’s ρ	Spearman r
Max	0.8071	0.9381
+ sigma	0.8959	0.9588
mean	0.9416	0.9392
- sigma	0.9591	0.9232
Min	0.9431	0.9202

**Table 4:** Correlation factors in the range from 973 kHz to 1.500 MHz for a motor with a 1 Ω resistance-in-series on a phase conductor.

Reference TF	Pearson’s ρ	Spearman r
Max	0.9228	0.9853
+ sigma	0.9900	0.9074
mean	0.9987	0.9988
- sigma	0.9953	0.9977
Min	0.8235	0.8103

**Table 5:** Correlation factors in the range from 100 kHz to 964 kHz for a motor with slight rotor misalignment.

Reference TF	Pearson’s ρ	Spearman r
Max	0.8676	0.8974
+ sigma	0.9289	0.9251
mean	0.9581	0.9733
- sigma	0.9655	0.9726
Min	0.9476	0.9698

**Table 6:** Correlation factors in the range from 964 kHz to 1.500 MHz for a motor with slight rotor misalignment.

Reference TF	Pearson’s ρ	Spearman r
Max	0.9197	0.9733
+ sigma	0.9919	0.9358
mean	0.9971	0.9901
- sigma	0.9930	0.9862
Min	0.8237	0.8103

**Table 7:** Correlation factors in the range from 100 kHz to 901 kHz for a motor with a slight rotor misalignment.

Reference TF	Pearson’s ρ	Spearman r
Max	0.8708	0.8831
+ sigma	0.9595	0.8938
mean	0.9467	0.9182
- sigma	0.9298	0.9426
Min	0.9143	0.9464

**Table 8:** Correlation factors in the range from 901 kHz to 1.500 MHz for a motor with a slight rotor misalignment.

Reference TF	Pearson’s ρ	Spearman r
Max	0.7186	0.6156
+ sigma	0.6963	0.7268
mean	0.6261	0.5844
- sigma	0.5786	0.5555
Min	0.2497	0.3677

**Table 9:** Correlation factors in the range from 100 kHz to 604 kHz of the motor after a stator fell from a height of 1 m.

Reference TF	Pearson’s ρ	Spearman r
Max	0.6116	0.6112
+ sigma	0.8207	0.7477
mean	0.9498	0.9444
- sigma	0.9644	0.9807
Min	0.9562	0.9832

**Table 10:** Correlation factors in the range from 604 kHz to 1.500 MHz of the motor after a stator fell from a height of 1 m.

Reference TF	Pearson’s ρ	Spearman r
Max	−0.4380	−0.4509
+ sigma	−0.6842	−0.6495
mean	−0.8160	−0.7434
- sigma	−0.8623	−0.7851
Min	−0.8498	−0.7992

**Table 11:** Correlation factors in the range from 100 kHz to 1.275 MHz for a motor with shorting ring damage.

Reference TF	Pearson’s ρ	Spearman r
Max	0.6329	0.8470
+ sigma	0.6429	0.8632
mean	0.7415	0.9265
-sigma	0.7877	0.9481
Min	0.8990	0.9739

**Table 12:** Correlation factors in the range from 1.275 MHz to 1.5 MHz for a motor with shorting ring damage.

Reference TF	Pearson’s ρ	Spearman r
Max	0.6352	0.6905
+ sigma	−0.8945	−1.000
mean	0.4729	0.4762
-sigma	0.5643	0.5953
Min	0.5169	0.5238

**Table 13:** Correlation factors in the range from 100 kHz to 568 kHz for a motor with rotor bar damage.

Reference TF	Pearson’s ρ	Spearman r
Max	0.6792	0.5475
+ sigma	0.8174	0.7012
mean	0.9865	0.9460
- sigma	0.9964	0.9900
Min	0.9852	0.9966

**Table 14:** Correlation factors in the range from 568 kHz to 1.5 MHz for a motor with rotor bar damage.

Reference TF	Pearson’s ρ	Spearman r
Max	−0.4198	−0.2950
+ sigma	−0.4361	−0.3223
mean	−0.5592	−0.5176
- sigma	−0.6133	−0.6213
Min	−0.7393	−0.7875

**Table 15:** Correlation factors in the range from 100 kHz to 1.275 MHz after the rotor group fell from a height of 1 m.

Reference TF	Pearson’s ρ	Spearman r
Max	0.5204	0.6854
+ sigma	0.5543	0.6978
mean	0.6668	0.8034
- sigma	0.7227	0.8330
Min	0.8318	0.8604

**Table 16:** Correlation factors in the range from 1.275 MHz to 1.5 MHz after the rotor group fell from a height of 1 m.

Reference TF	Pearson’s ρ	Spearman r
Max	0.6366	0.6905
+ sigma	-0.8950	-1.000
mean	0.4730	0.4762
- sigma	0.5645	0.5952
Min	0.5146	0.5238

**Table 17:** Correlation factors in the range from 100 kHz to 1.300 MHz for a motor with a shortcut between 2 winding turns.

Reference TF	Pearson’s ρ	Spearman r
Max	0.4918	0.8303
+ sigma	0.4696	0.8127
mean	0.5731	0.8391
- sigma	0.6252	0.8652
Min	0.7673	0.8974

**Table 18:** Correlation factors in the range from 1.300 MHz to 1.500 MHz for a motor with a shortcut between 2 winding turns.

Reference TF	Pearson’s ρ	Spearman r
Max	0.5297	0.5357
+ sigma	−0.8363	−1.000
mean	0.1609	0.2143
- sigma	0.3560	0.3929
Min	0.2523	0.2857

## Data Availability

Not applicable.
